# Clinical difficulties in the treatment of restless legs syndrome: it is the dose that makes poison

**DOI:** 10.1192/j.eurpsy.2022.1867

**Published:** 2022-09-01

**Authors:** S. Petrykiv, M. Arts, L. Jonge

**Affiliations:** 1 GGZWNB, Psychiatry, Halsteren, Netherlands; 2 GGZWNB, Psychiatry, Bergen op Zoom, Netherlands

**Keywords:** Ropinirole, Depression, Side effects, Restless legs syndrome

## Abstract

**Introduction:**

Polyfarmacy and unjustified use of high dosages of medicaments represent an unmet need in modern psychiatry. Therefore, tidal medication review of hospitalized geriatric patients is an essential step of the disease management as it can be often of vital importance and, as illustrated by current case, can exhibit a tremendeus impact on their quality of life.

**Objectives:**

A case rapport on geriatric patient with iatrogenic damage due to ultra high dosage of ropinirole as a treatment for restless legs syndrome

**Methods:**

Authors of current paper address pharmacodynamic particularities of psychopharmaca and their reasonable choice in context to RLS

**Results:**

A clinical case of a 72 y.o. patient, known with chronic minor depressive symptomes over the past decades. Since few years he did not take any medicaion, except ropinirol for RLS. Because of the worsening of RLS symptomes, he decided on his own to increase the dose of ropinirol up to 12 mg/day. Two moths later he has been admitted to the psychiatric ward with major depression symptomes, suicidal plans, insomnia and profound edema of his both lower legs.

**Conclusions:**

Current case demonstrates that high dose of ropinirole led to tremendous decrease of quality of life of the patient, and pushed him towards concrete suicidal plans. We advocate for careful assessing of the dose of every drug used; avoiding of polypharmacy by any means and for keeping in consideration that the majority of psychopharmaca leads to deterioration of RLS symptoms through modulation of dopamine pathways.

**Disclosure:**

No significant relationships.

